# Constitutive NF-***κ***B Activation Underlines Major Mechanism of Drug Resistance in Relapsed Refractory Diffuse Large B Cell Lymphoma

**DOI:** 10.1155/2015/484537

**Published:** 2015-04-23

**Authors:** Francesco Turturro

**Affiliations:** Department of Lymphoma/Myeloma, MD Anderson Cancer Center, Unit 429, 1515 Holcombe Boulevard, Houston, TX 77030, USA

## Abstract

Diffuse large B cell lymphoma (DLBCL) is the most common subtype of B cell non-Hodgkin's lymphoma (NHL), encompassing 30–40% of the estimated 70,000 cases of NHL in 2014 in the USA. Despite major improvements with immune-chemotherapy, the fraction of patients who still succumb to a refractory or relapsed disease remains high. This review addresses whether the better understanding of the biology of DLBCL defines new therapeutic avenues that may overcome the emerging resistance of this disease to traditional immune-chemotherapy, such as rituximab in combination with traditional chemotherapy agents. Emerging targeted therapy for relapsed refractory DLBCL encompasses more complex molecular abnormalities involving signaling pathways other than NF-*κ*B as mechanism of resistance to immune-chemotherapy. Our review suggests that NF-*κ*B pathway is an important crossroad where other pathways converge as phenotype of resistance that emerges in patients who fail frontline and salvage immune-chemotherapy. Future efforts should aim at targeting the role of NF-*κ*B resistance in clinical trials, where novel agents like lenalidomide and proteasome inhibitors with established activity in this perspective will be an important component in combination therapy, along with new monoclonal antibody, BTK-inhibitors, and other novel therapy agents.

## 1. Introduction

Diffuse large B cell lymphoma (DLBCL) is the most common subtype of B cell non-Hodgkin's lymphoma (NHL), encompassing 30–40% of the estimated 70,000 cases of NHL in 2014 in USA [1]. Although DLBCL is potentially curable, 30–40% of patients eventually relapse or are primarily refractory and fail to achieve complete remission (CR). Nearly 19,000 affected subjects are projected to die of DLBCL in 2014. As consequence of this incidence, even slight improvement in the outcomes of DLBCL patients has the potential for high impact on their survival, making the study of combination of novel drugs paramount. Historically, the combination regimen CHOP (cyclophosphamide, doxorubicin, vincristine, prednisone) established itself as standard of care in a landmark randomized clinical trial in 1993 [2]. Later the “spinoff regimen” CHOP-derivative DA-EPOCH revealed increased efficacy particularly for high grade B-cell lymphoma by modifying the infusion regimen and by adding etoposide [3]. Finally, the addition of targeted therapy with the anti-CD20 monoclonal antibody rituximab has further improved the outcomes of patients with DLBCL [4–6]. Despite major improvements with immune-chemotherapy, the fraction of patients who still succumb to a refractory or relapsed disease remains high. This review will address whether the better understanding of the biology of DLBCL may define new therapeutic avenues that overcome the resistance of this disease to traditional immune-chemotherapy.

## 2. Heterogeneity of DLBCL

DLBCL revealed itself as a disease more heterogeneous than the 2008 WHO classification had initially defined [7]. In fact, recent studies have established that DLBCL is associated with various genetic alterations and high biologic diversity. This has been studied [8]. The cell of origin (COO) approach based on gene expression profiling (GEP) of DLBCL has defined molecularly the disease better than the morphology. The prognostic value of COO approach has been investigated and compared to the International Prognostic Index (IPI) in patients treated with anthracycline-based regimens and rituximab [9]. In fact based on retrospective analysis, patients with germinal center B (GCB) DLBCL type have a better prognosis of patients with activated B cell (ABC) type when treated with R-CHOP or R-DA-EPOCH [10, 11], independently of the IPI. Therefore, patients who relapse or are refractory to initial therapy are more likely to belong to the ABC type. For the same reason, ABC type is expected to represent the majority of patients who have poorly responded to salvage chemotherapy like R-DHAP or R-ICE as shown prospectively in the CORAL study [12]. A recent retrospective study has shown that 30% of patients with DLBCL treated with second line therapy are truly nonresponders [13]. In this population immune-chemotherapy-based third line therapy yields a response rate (RR) of 20% with median overall survival (OS) of only 4 months [13]. Furthermore, patients who have relapsed after autologous stem cell transplant have median OS of 9 months [14]. In this group, patients who received novel agents (lenalidomide, non-rituximab monoclonal antibodies, tyrosine kinase inhibitors, and radioimmunotherapy) had a longer median OS of 11.3 months as compared to 6.6 months for patients treated with conventional cytotoxic chemotherapy [14].

## 3. Relevance and Heterogeneity of the Constitutive NF-*κ*B Activation in DLBCL

The NF-*κ*B family of proteins is a group of transcription factors (RelA, RelB, c-Rel, NF-*κ*B1, and NF-*κ*B2) that are kept inactive by a group of inhibitory cytoplasmic proteins, the I*κ*B kinase complex [15, 16]. The molecular hallmark of the ABC-type of DLBCL is the constitutive pathogenic activation of the nuclear factor-*κ*B (NF-*κ*B) pathway to which several mechanisms converge to ultimately promote cell proliferation and protection from apoptosis [8]. Three proteins—CARD11, BCL10, and MALT1—form a signaling complex (CBM) leading to the activation of NF-*κ*B pathway following antigen stimulation of the B cell receptor (BCR) [17]. Furthermore, MYD88 encodes an adaptor protein that activates NF-*κ*B and JAK2/STAT3 signaling pathways through stimulation of the toll-like and interleukin receptors [18, 19]. The driver-nature of the mutations affecting all the mentioned genes leads to gain-of-function that promotes cell survival and prevents apoptosis [18]. A recent study has established that activating mutations involving at least one of the four genes involving the NF-*κ*B pathway (MYD88, CD79A/B, and CARD11) are present in 30–40% of 161 patients affected with DLBCL, independently from their COO phenotype [20]. These mutations are distributed with at least one mutation present in 39% of patients with the ABC, 23% with the GCB, and 23% in the indeterminate group of the COO phenotype [20]. Interestingly, patients harboring at least 1 mutation targeting one of the four genes of the NF-*κ*B pathway “mutated” as compared to patients in the ABC-DLBCL group without mutations “unmutated” had a 3-year overall survival (OS) of 66.7% versus 26.1%, respectively (*P* = 0.0337) [20].

Although historically it has been recognized that NF-*κ*B pathway is engaged in ABC-type DLBCL through chronic active BCR signaling, a recent work has shown that the members of the NF-*κ*B transcription factors (NF-*κ*B1, NF-*κ*B2, RELA, RELB, and REL) are expressed in 88% of tissue derived from both ABC and GCB type of DLBCL [21]. In this study, the tissues from 188 patients with DLBCL were evaluated by immunohistochemistry (IHC) and validated in a subset with gene expression profiling. Furthermore, no significant differences regarding the expression of the different NF-*κ*B family of transcription factors were detected between the two COO subtypes. This suggests that upregulation of NF-*κ*B signaling pathway is of relevance not only in the ABC subtype, but also in the GCB counterpart [21]. Odqvist et al. made the case for NF-*κ*B pathway to be more broadly engaged in DLBCL than initially estimated and potentially responsible for the resistance to the traditional cytotoxic immunochemotherapy. This study also established that REL expression has a significant favorable clinical impact in patient treated with R-CHOP and identified a subgroup of patients with superior outcome (5-year OS of 73.7% versus 59.7% in REL-positive and REL-negative patients, resp.; *P* = 0.0041) [21]. This finding may be relevant in defining prospectively patients with favorable versus unfavorable prognosis in response to frontline R-CHOP, based on the expression of the members of the NF-*κ*B family of transcription factors. To further support our hypothesis that has driven the current review, more work has been recently published favoring the role of NF-*κ*B pathway as mechanism of resistance. Gene expression profiling in tissue prospectively collected from 51 patients with DLBCL treated with immunochemotherapy has identified 31 genes whose expression changes were strongly associated with copy number aberrations or gains of chromosome 2p15 and 18q12.2 and unfavorable survival [22]. The 2p15 abnormality (amplification) that harbors the gene COMMD1 and expression of the COMMD1 protein by IHC were associated with inferior progression free survival (PFS) as compared to patients without the amplification (*P* = 0.010 and *P* = 0.003, resp.) [22]. COMMD family of proteins plays a distinct and nonredundant role in NF-*κ*B signaling [23]. More recently, it has been shown that NR4A1 (Nur77) expression was significantly associated with poor survival in patients with aggressive large B-cell lymphoma [24].* In vitro *overexpression of this putative tumor suppressor gene induced apoptosis in lymphoma cells [24]. Previous work had shown that NR4A1 (Nur77) blocks NF-*κ*B activation [25]. More interesting, NF-*κ*B signaling pathway is activated in EBV-positive DLBCL in both the elderly and nonelderly de novo DLBCL, and it seems to have an impact on the outcome of this patient population [26, 27]. Since MYD88 L265P mutation is a hallmark of lymphoma with lymphoplasmacytic features and activated NF-*κ*B signaling pathway, the higher frequency of MYD88 mutations in the ABC phenotype suggests that this subtype may derive from cells with those features. Furthermore, the presence of serum IgM-paraprotein is more frequently associated with lymphoma with lymphoplasmacytic features. It is tempting to speculate that DLBCL patients who present with immunoblastic features and associated serum monoclonal IgM may have a higher frequency of MYD88 mutations as a hallmark of a subtype of ABC phenotype. None of the patients with increased levels of IgM was harboring MYD88 mutation [28]. Although NF-*κ*B signaling pathway was not studied in those patients with associated monoclonal IgM, they responded to a combination of bortezomib and lenalidomide, active agents in patients with activated NF-*κ*B signaling pathway [28]. This study suggested that the presence of monoclonal IgM or elevated free heavy IgM chains was associated with favorable response to inhibitors of the NF-*κ*B signaling pathway independently from the presence of MYD88 mutation [29, 30].

## 4. Pharmacological Attempts to Overcome the Constitutive NF-*κ*B Activation-Mediated Resistance with Novel Agents in DLBCL

The proteasome is an intracellular, multiunit-protease complex that regulates protein degradation and remodeling. Bortezomib, first-in-class drug approved for multiple myeloma and relapsed/refractory mantle zone lymphoma (MCL), binds to the *β*-subunits of the core of the proteasome and inactivates NF-*κ*B by stabilization of the NF-*κ*B-inhibitor I*κ*B kinase complex [31]. Despite its inhibition of the NF-*κ*B pathway, single agent bortezomib has shown little clinical activity in patients with DLBCL [19]. The addition of bortezomib to R-CHOP in untreated patients with DLBCL resulted in overall response rate of 89% in one study and 86% in another [32, 33]. Although a study of bortezomib combined with EPOCH in patients with relapsed or refractory DLBCL showed overall modest activity, ABC type seemed to benefit dramatically more than CGB type (RR 83% versus 13%, *P* < 0.01, and median OS 10.8 versus 3.4 months, *P* = 0.003) [22]. The PYRAMID study is evaluating prospectively R-CHOP with and without bortezomib in untreated patients with DLBCL [34]. Previously, a phase I/II study of bortezomib with gemcitabine for relapsed or refractory DLBCL showed a very modest RR of 10% [35]. Ixazomib (formerly known as MLN9708) is a selective, orally bioavailable, second-generation proteasome inhibitor that has shorter proteasome dissociation half-life and improved pharmacokinetics, pharmacodynamics, and antitumor activity compared with bortezomib [36]. Furthermore, MLN9708 has a larger blood volume distribution at steady state and greater pharmacodynamics effects in tissue than bortezomib. Finally, MLN9708 showed activity in hematologic preclinical xenograft models and increased correlation between pharmacodynamics responses and improved antitumor activity [36]. To date, MLN9708 in its intravenous (IV) formulation has only been studied in a phase I dose escalation of once weekly in patients with relapsed or refractory follicular lymphoma (FL) and peripheral T-cell lymphoma (PTCL), but not in patients with DLBCL [37].

DLBCL ABC subtype presents with recurrent oncogenic mutations activating both the B-cell receptor (BCR) and MYD88 pathways for driving the NF-*κ*B pathway and favoring cell survival [17]. MYD88 signaling pathway also induces IFN*β*, detrimental to ABC-DLBCL survival. The complex IRF4/SPIB sits at the crossroad of the two pathways and promotes ABC-DLBCL survival with interaction with by IRF7, IFN*β*, and trans-activation of CARD11 that results in increased NF-*κ*B signaling activity. NF-*κ*B factors transactivate IRF4 by a positive feedback oncogenic loop. Lenalidomide, an immunomodulatory drug (IMID), with activity in B-cell non-Hodgkin's lymphoma (NHL), targets this circuit by downregulating IRF4/SBIP, increasing toxic IFN*β* secretion, and decreasing NF-*κ*B activity [17, 38]. In fact, lenalidomide as single agent has shown RR of 35% in 49 patients with relapsed/refractory aggressive NHL [39]. RR was 33% (17 patients) and 41% (45 patients) in two other studies of lenalidomide in combination with rituximab for the treatment of relapsed/refractory DLBCL [40, 41].

The convenience of the availability of an oral agent like MLN9708, with better pharmacokinetic and pharmacodynamic profile than bortezomib, makes this second generation proteasome inhibitor a better candidate for studying it in combination with lenalidomide. The combination of two oral agents will make more convenient the use of MLN9708 and lenalidomide in a population of patient with rituximab resistance. The CORAL study has shown that patients with DLBCL exposed to rituximab as part of the initial therapy had inferior response to salvage therapy versus patients who did not receive rituximab (51% versus 83%, *P* < 0.001), supporting the hypothesis that those patients may have acquired resistance to rituximab [12]. It has been shown that NF-*κ*B signaling pathway modulates the response to rituximab and chemosensitization of the NHL B-cell [42]. Finally, a recent article has shown that combined lenalidomide, low dose dexamethasone, and rituximab overcome the rituximab resistance in patients with indolent lymphoma and MCL [43]. In this context, an oral combination therapy targeting dysregulation of NF-*κ*B may be more effective in a population of DLBCL refractory to salvage immune-chemotherapy with R-DHAP, RICE, or similar cisplatin-Ifosfamide-etoposide-based regimens, particularly in view of the most recent data [44]. In fact, only 44% (64/145) of patients were able to be transplanted after crossing over RICE/R-DHAP as 3rd line of therapy [44].

Certainly, the biology of relapsed/refractory DLBCL is complex and involves molecular abnormalities other than NF-*κ*B as mechanism of resistance to immune-chemotherapy that are not covered in this review [8]. We have shown that NF-*κ*B signaling pathway seems to be at an important crossroad where other pathways converge as “resistance-phenotype” in patients who fail frontline and salvage immune-chemotherapy. In this context, lenalidomide and new generation proteasome inhibitors may represent a new platform in combination with new monoclonal antibody, BTK inhibitors, and other novel therapy agents in future studies [8, 45].

## References

[1] Siegel R., Ma J., Zou Z., Jemal A. (2014). Cancer statistics, 2014. *CA: A Cancer Journal for Clinicians*.

[2] Fisher R. I., Gaynor E. R., Dahlberg S. (1993). Comparison of a standard regimen (CHOP) with three intensive chemotherapy regimens for advanced non-Hodgkin's lymphoma. *The New England Journal of Medicine*.

[3] Wilson W. H., Grossbard M. L., Pittaluga S. (2002). Dose-adjusted EPOCH chemotherapy for untreated large B-cell lymphomas: a pharmacodynamic approach with high efficacy. *Blood*.

[4] Coiffier B., Lepage E., Brière J. (2002). Chop chemotherapy plus rituximab compared with chop alone in elderly patients with diffuse large-B-cell lymphoma. *The New England Journal of Medicine*.

[5] Coiffier B., Thieblemont C., van den Neste E. (2010). Long-term outcome of patients in the LNH-98.5 trial, the first randomized study comparing rituximab-CHOP to standard CHOP chemotherapy in DLBCL patients: a study by the Groupe d'Etudes des Lymphomes de l'Adulte. *Blood*.

[6] Pfreundschuh M., Kuhnt E., Trümper L. (2011). CHOP-like chemotherapy with or without rituximab in young patients with good-prognosis diffuse large-B-cell lymphoma: 6-year results of an open-label randomised study of the MabThera International Trial (MInT) Group. *The Lancet Oncology*.

[7] Alizadeh A. A., Elsen M. B., Davis R. E. (2000). Distinct types of diffuse large B-cell lymphoma identified by gene expression profiling. *Nature*.

[8] Roschewski M., Staudt L. M., Wilson W. H. (2014). Diffuse large B-cell lymphoma—treatment approaches in the molecular era. *Nature Reviews Clinical Oncology*.

[9] Fu K., Weisenburger D. D., Choi W. W. L. (2008). Addition of rituximab to standard chemotherapy improves the survival of both the germinal center B-cell-like and non-germinal center B-cell-like subtypes of diffuse large B-cell lymphoma. *Journal of Clinical Oncology*.

[10] Wilson W. H., Dunleavy K., Pittaluga S. (2008). Phase II study of dose-adjusted EPOCH and rituximab in untreated diffuse large B-cell lymphoma with analysis of germinal center and post-germinal center biomarkers. *Journal of Clinical Oncology*.

[11] Wilson W. H., Jung S.-H., Porcu P. (2012). A cancer and Leukemia Group B multi-center study of DA-EPOCH-rituximab in untreated diffuse large B-cell lymphoma with analysis of outcome by molecular subtype. *Haematologica*.

[12] Gisselbrecht C., Glass B., Mounier N. (2010). Salvage regimens with autologous transplantation for relapsed large B-cell lymphoma in the rituximab era. *Journal of Clinical Oncology*.

[13] Elstrom R. L., Martin P., Ostrow K. (2010). Response to second-line therapy defines the potential for cure in patients with recurrent diffuse large B-cell lymphoma: implications for the development of novel therapeutic strategies. *Clinical Lymphoma, Myeloma and Leukemia*.

[14] Nagle S. J., Woo K., Schuster S. J. (2013). Outcomes of patients with relapsed/refractory diffuse large B-cell lymphoma with progression of lymphoma after autologous stem cell transplantation in the rituximab era. *American Journal of Hematology*.

[15] Nagel D., Vincendeau M., Eitelhuber A. C., Krappmann D. (2014). Mechanisms and consequences of constitutive NF-*κ*B activation in B-cell lymphoid malignancies. *Oncogene*.

[16] Lam L. T., Davis R. E., Pierce J. (2005). Small molecule inhibitors of I*κ*B kinase are selectively toxic for subgroups of diffuse large B-cell lymphoma defined by gene expression profiling. *Clinical Cancer Research*.

[17] Yang Y., Shaffer A. L., Emre N. C. T. (2012). Exploiting synthetic lethality for the therapy of ABC diffuse large B cell Lymphoma. *Cancer Cell*.

[18] Compagno M., Lim W. K., Grunn A. (2009). Mutations of multiple genes cause deregulation of NF-*κ*B in diffuse large B-cell lymphoma. *Nature*.

[19] Ngo V. N., Young R. M., Schmitz R. (2011). Oncogenically active MYD88 mutations in human lymphoma. *Nature*.

[20] Bohers E., Mareschal S., Bouzelfen A. (2014). Targetable activating mutations are very frequent in GCB and ABC diffuse large B-cell lymphoma. *Genes Chromosomes and Cancer*.

[21] Odqvist L., Montes-Moreno S., Sánchez-Pacheco R. E. (2014). NF*κ*B expression is a feature of both activated B-cell-like and germinal center B-cell-like subtypes of diffuse large B-cell lymphoma. *Modern Pathology*.

[22] Taskinen M., Louhimo R., Koivula S. (2014). Deregulation of COMMD1 is associated with poor prognosis in diffuse large B-cell lymphoma. *PLoS ONE*.

[23] Bartuzi P., Hofker M. H., van de Sluis B. (2013). Tuning NF-*κ*B activity: a touch of COMMD proteins. *Biochimica et Biophysica Acta—Molecular Basis of Disease*.

[24] Deutsch A. J. A., Rinner B., Wenzl K. (2014). NR4A1-mediated apoptosis suppresses lymphomagenesis and is associated with a favorable cancer-specific survival in patients with aggressive B-cell lymphomas. *Blood*.

[25] Harant H., Lindley I. J. D. (2004). Negative cross-talk between the human orphan nuclear receptor Nur77/NAK-1/TR3 and nuclear factor-*κ*B. *Nucleic Acids Research*.

[26] Montes-Moreno S., Odqvist L., Diaz-Perez J. A. (2012). EBV-positive diffuse large B-cell lymphoma of the elderly is an aggressive post-germinal center B-cell neoplasm characterized by prominent nuclear factor-*κ*B activation. *Modern Pathology*.

[27] Ok C. Y., Li L., Xu-Monette Z. Y. (2014). Prevalence and clinical implications of Epstein-Barr virus infection in de Novo diffuse large B-cell lymphoma in western countries. *Clinical Cancer Research*.

[28] Cox M. C., Di Napoli A., Scarpino S. (2014). Clinicopathologic characterization of diffuse-large-B-cell lymphoma with an associated serum monoclonal IgM component. *PLoS ONE*.

[29] Ruminy P., Etancelin P., Couronné L. (2011). The isotype of the BCR as a surrogate for the GCB and ABC molecular subtypes in diffuse large B-cell lymphoma. *Leukemia*.

[30] Jardin F., Delfau-Larue M. H., Molina T. J. (2013). Immunoglobulin heavy chain/light chain pair measurement is associated with survival in diffuse large B-cell lymphoma. *Leukemia & Lymphoma*.

[31] Mato A. R., Feldman T., Goy A. (2012). Proteasome inhibition and combination therapy for non-Hodgkin's lymphoma: from bench to bedside. *The Oncologist*.

[32] Ruan J., Martin P., Furman R. R. (2011). Bortezomib plus CHOP-rituximab for previously untreated diffuse large B-cell lymphoma and mantle cell lymphoma. *Journal of Clinical Oncology*.

[33] Mounier N., Ribrag V., Haioun C. (2007). Efficacy and toxicity of two schedules of R-CHOP plus bortezomib in front-line B lymphoma patients. A randomized phase II trial from the Groupe d’Etude des Lymphomes de l’Adulte (GELA). *Journal of Clinical Oncology*.

[34] Doner K., Flinn I. W., Ulrich B. K. (2010). Rapid prospective identification of non-germinal center B cell-like (GCB) diffuse large B-cell lymphoma (DLBCL) patients for targeted trials: Early results from PYRAMID, a phase 2 randomized study of R-CHOP +/- bortezomib in newly diagnosed non-GCB DLBCL. *Blood*.

[35] Evens A. M., Rosen S. T., Helenowski I. (2013). A phase I/II trial of bortezomib combined concurrently with gemcitabine for relapsed or refractory DLBCL and peripheral T-cell lymphomas. *British Journal of Haematology*.

[36] Kupperman E., Lee E. C., Cao Y. (2010). Evaluation of the proteasome inhibitor MLN9708 in preclinical models of human cancer. *Cancer Research*.

[37] Assouline S., Chang J. E., Cheson B. D. (2012). Results of a Phase 1 dose-escalation study of once-weekly MLN9708, an investigational proteasome inhibitor, in patients with relapsed/refractory lymphoma. *Blood*.

[38] Zhang L.-H., Kosek J., Wang M., Heise C., Schafer P. H., Chopra R. (2013). Lenalidomide efficacy in activated B-cell-like subtype diffuse large B-cell lymphoma is dependent upon IRF4 and cereblon expression. *British Journal of Haematology*.

[39] Wiernik P. H., Lossos I. S., Tuscano J. M. (2008). Lenalidomide monotherapy in relapsed or refractory aggressive non-Hodgkin's lymphoma. *Journal of Clinical Oncology*.

[40] Ivanov V., Coso D., Chetaille B. (2014). Efficacy and safety of lenalinomide combined with rituximab in patients with relapsed/refractory diffuse large B-cell lymphoma. *Leukemia & Lymphoma*.

[41] Wang M., Fowler N., Wagner-Bartak N. (2013). Oral lenalidomide with rituximab in relapsed or refractory diffuse large cell, follicular and transformed lymphoma: a phase II clinical trial. *Leukemia*.

[42] Jazirehi A. R., Huerta-Yepez S., Cheng G., Bonavida B. (2005). Rituximab (chimeric anti-CD20 monoclonal antibody) inhibits the constitutive nuclear factor-*κ*B signaling pathway in non-Hodgkin's lymphoma B-cell lines: role in sensitization to chemotherapeutic drug-induced apoptosis. *Cancer Research*.

[43] Ahmadi T., Chong E. A., Gordon A. (2014). Combined lenalidomide, low-dose dexamethasone, and rituximab achieves durable responses in rituximab-resistant indolent and mantle cell lymphomas. *Cancer*.

[44] Van Den Neste E., Gisselbrecht C., Schmitz N. (2013). Diffuse large B-cell lymphoma (DLBCL) patients failing second-line R-DHAP or R-ICE chemotherapy included in the Coral Study. *Blood*.

[45] Barton S., Hawkes E. A., Wotherspoon A., Cunningham D. (2012). Are we ready to stratify treatment for diffuse large B-cell lymphoma using molecular hallmarks?. *Oncologist*.

